# Persistent CSF leak post spinal surgery and cerebrospinal fluid dynamic disturbances: cause or consequence?

**DOI:** 10.1186/2045-8118-12-S1-P11

**Published:** 2015-09-18

**Authors:** Claudia Craven, Neekhil A Patel, Akbar A Khan, Simon D Thompson, Edward W Dyson, Samir A Matloob, Aswin Chari, Patricia Haylock-Vize, Syed N Shah, Andrew R Stevens, Tarek Mostafa, Huan Wee Chan, Jinendra Ekanayake, Ahmed K Toma, Laurence D Watkins

**Affiliations:** 1 Victor Horsley Department of Neurosurgery, National Hospital for Neurology and Neurosurgery, Queen Square, London, UK

## Introduction

Cerebrospinal fluid (CSF) leak following spinal surgery is a relatively common surgical complication. A small group of CSF leak patients require multiple surgical repairs and prolonged hospital admission. Spinal CSF leaks are usually classically associated with symptoms of low intracranial pressure (ICP). However, there is a paucity of literature investigating the associated CSF dynamics.

## Methods

Retrospective case series study of patients with persistent CSF leak referred to the hydrocephalus service in our unit for intracranial pressure monitoring. Medical notes were reviewed for clinical presentation, management and outcome. Images were reviewed and ICP data were analysed. All patients underwent Continuous ICP monitoring using Spiegelberg ICP bolts.

## Results

3 Patients had cervical, thoracic and lumbosacral complex spinal fixation surgery, complicated by prolonged CSF leaks (mean of 56 days from day of surgery to resolution). Each patient required 2 re-explorations spinal surgeries and multiple lumbar drains insertions prior to 24 hours ICP monitoring. Two patients were shown to have mildly raised ICP (>15.2mmHg) and all three had abnormal pulse amplitudes (>5mmHg). One patient underwent catheter cerebral venogram that demonstrated focal stenosis of the distal right transverse sinus with a significant pressure gradient. This patient underwent a right transverse venous sinus stent insertion. This resulted in resolution of headaches, prevention of impending wound breakdown and normalisation of ICP data. Two patients underwent insertion of ventriculo-peritoneal shunts (VPS). Both had resolution of their CSF leaks immediately post VPS insertion and were discharged from hospital.

## Conclusion

Our results suggest that abnormal cerebrospinal fluid dynamics should be explored in patients with persistent CSF leak post spinal surgery. Whether abnormal pressure and dynamics represent a pre-existing abnormality or is induced by spinal surgery should be subject for further studies.

## Abbreviations

ICP: Intra Cranial Pressure; VPS: Ventriculo-peritoneal shunt.

